# Prognostic and immunological landscape of DDX17 in pan-cancer analysis: a comprehensive study

**DOI:** 10.1007/s12672-025-02955-9

**Published:** 2025-06-17

**Authors:** Yu Lei, Xin He, Peng Chen, Yiping Qin, Bin Ge, Yan Liu, Pu Li, Xing Wei

**Affiliations:** 1 Department of Clinical Laboratory, Pidu District People’s Hospital, Third Affiliated Hospital of Chengdu Medical College, Chengdu, 611730 Sichuan People’s Republic of China; 2 Department of Clinical Laboratory, School of Medicine, Chongqing University Jiangjin Hospital, Chongqing University, Jiangjin, 402260 Chongqing People’s Republic of China; 3 Chongqing Key Laboratory of Translational Research for Cancer Metastasis and Individualized Treatment, Chongqing University Cancer Hospital, Chongqing, 400030 People’s Republic of China

**Keywords:** DDX17, Pan-cancer, Prognosis, Immune infiltration, Immunotherapy, Biomarker

## Abstract

**Objective:**

DDX17, an ATP-dependent RNA/DNA helicase, is implicated in the regulation of RNA metabolism and has been linked to tumorigenesis and metastasis in various cancers. While studies have explored the role of DDX17 in specific cancers, further research is needed to understand its mechanisms across different cancer types.

**Methods:**

We leveraged several public databases, including TIMER, The Cancer Genome Atlas (TCGA), and the Genotype-Tissue Expression (GTEx), to investigate DDX17 mRNA expression across 33 types of tumors. The GEPIA2 database was utilized to assess the impact of DDX17 on overall survival (OS) and disease-free survival (DFS) in patients with these tumors. Additionally, we employed cBioPortal to examine DDX17 gene alterations in various tumor tissues. Further analysis was conducted using the R language to explore the correlation between DDX17 and a range of clinical features, including tumor microenvironment (TME), immune regulatory genes, immune checkpoints, tumor mutational burden (TMB), microsatellite instability (MSI), DNA methylation, RNA methylation, and drug sensitivity. Gene Set Enrichment Analysis (GSEA) was also applied to elucidate the molecular mechanisms mediated by DDX17.

**Results:**

DDX17 showed significant differences in expression between cancer and normal tissues. Expression of DDX17 was associated with patient prognosis, TMB, MSI, and drug sensitivity in certain cancers. DDX17 is additionally involved in modulating immune functions and influencing the tumor microenvironment. Further analysis of DDX17 mutation sites and types showed that the mutation frequency was highest in endometrial cancer and the major mutations of DDX17 were missense mutations.

**Conclusion:**

These findings indicated that DDX17 may be considered a potential prognostic biomarker and a promising target for novel immunotherapeutic approaches in cancer treatment.

**Supplementary Information:**

The online version contains supplementary material available at 10.1007/s12672-025-02955-9.

## Introduction

The majority of nations worldwide now recognize cancer as a significant public health threat [1]. According to the Global Cancer Observatory 2022 report, in 2020, there were 19,292,789 new cases of cancer and 9,958,133 cancer-related deaths globally [2]. The burden of global cancer is projected to escalate further. One study unveiled an anticipated surge of 49% in new cancer cases and a 62% increase in cancer fatalities [3]. Despite the increasing availability of conventional cancer treatments, millions of individuals succumb to this disease annually [4]. Recent research has elucidated the intricate association between tumor occurrence, progression, recurrence, and metastasis with the tumor microenvironment (TME) [5]. Tumor immunity plays a pivotal role in tumor recurrence and an unfavorable prognosis, hence it is imperative to investigate its underlying mechanisms.

The DEAD-box (DDX) protein family represents the largest group of RNA helicases, encompassing enzymes responsible for unwinding double-stranded RNA. Members of the DDX protein family are characterized by a conserved D-E-A-D amino acid motif, which is composed of glutamic acid (Glu, E), alanine (Ala, A), and aspartic acid (Asp, D) [6]. Previous studies have shown that DDX family genes play a crucial role in prostate cancer, papillary thyroid carcinoma, and liver cancer [7]. DDX17, a prototypical member of the DEAD-box family, is an ATP-dependent RNA/DNA helicase with multifaceted functions in regulating RNA metabolism [7]. Aberrant expression of DDX17 has been observed in various tumor types, and it plays a pivotal role in tumorigenesis, as well as cancer cell proliferation, invasion, and metastasis through intricate mechanisms such as RNA binding, transcriptional regulation, and microprocessor complex formation [8]. These processes exert profound influences on the trajectory of cancer development. A study demonstrated that DDX17 promotes epithelial-mesenchymal transition and metastasis via the miR-149-3p/CYBRD1 pathway in colorectal cancer (CRC) patients, which significantly contributes to aggressive progression and prognosis of CRC [9]. Chen’s group confirmed that DDX17 acts as a pro-tumor splicing factor during hepatocellular carcinoma (HCC) progression and identified it as an oncoprotein [10]. Furthermore, DDX17 has been shown to play a regulatory role in tumor progression across diverse cell types including breast cancer, non-small cell lung cancer, and glioma [11–13]. Additionally, DDX17 participates in the YAP signaling pathway to increase the stemness of cancer stem-like cells and to promote tumorigenesis and tumor progression [14].

However, current research on DDX17 primarily focuses on a limited number of cancer types, and its roles in other tumors remain to be further explored. To comprehensively assess the role of DDX17 in cancer, we conducted an integrated analysis of data from various databases including TCGA, Genotype-Tissue Expression Project (GTEx), cBioPortal, and Human Protein Atlas (HPA), examining the expression patterns of DDX17 and its correlations with clinical survival, immune checkpoints, prognostic value, immune modulators, genomic profiles, immune characteristics as well as DNA and RNA methylation status. Our aim is to reveal the broad prospects of DDX17 as a prognostic biomarker and therapeutic intervention target through systematic analysis across different cancer types.

## Materials and methods

### The analysis of the localization and expression levels of DDX17

To analyze the differences in DDX17 expression between cancerous and normal tissues, we obtained cancer transcriptome data from the University of California, Santa Cruz (UCSC) Xena database (https://xenabrowser.net/). The abbreviations of 33 types of tumors are shown in Table S1. All data were then normalized using a log2(x + 0.001) transformation for further analysis [15, 16]. Additionally, we utilized the Gene Expression Profiling Interactive Analysis (GEPIA2, http://gepia.cancer-pku.cn/) database to examine the correlation between DDX17 expression and clinicopathological staging [16]. This advanced server is capable of analyzing RNA sequencing data effectively.

### Pathological stages analysis

The correlation between DDX17 expression and tumor stage was analyzed using the “Stage plot” function in GEPIA2. The dataset used for this analysis was TCGA Pan-Cancer (PANCAN, *N* = 10,535, G = 60,499), obtained from the UCSC database. Expression data of the DDX17 were extracted from each sample after excluding samples with expression levels of 0, this ensured the reliability of the analysis while maintaining a representative sample size. Although this reduced the total number of samples, it minimized potential biases introduced by incomplete or unreliable data. To normalize the expression values, a log2(x + 0.001) transformation was applied. Cancer types with less than 3 samples per type were excluded to ensure robustness, resulting in expression data for 37 cancer types. Differential expression analysis of genes across different clinical stages within each tumor was performed using R software (version 3.6.4). Non-paired Student’s t-test was used for pairwise significance analysis of differences, while analysis of variance was employed for differential testing of multiple sample groups [15].

### Prognosis analysis

We employed GEPIA2 to obtain the significance map data and survival plots of DDX17 across all TCGA tumors for Overall Survival (OS) and Disease-Free Survival (DFS). The high and low expression cohorts were separated using cutoff thresholds at 50%. To assess statistical significance, we conducted hypothesis testing using the log-rank test. The pan-cancer dataset, TCGA Pan-Cancer, was sourced from the UCSC database. Expression data of the DDX17 was extracted from each sample while excluding samples with zero expression levels or follow-up times shorter than 30 days. A logarithmic transformation was applied to normalize the expression values, incorporating a pseudo-count of 0.001. Additionally, cancer types with less than 10 samples per type were excluded from analysis. While this exclusion may introduce a potential bias toward patients with longer follow-up, it reduces the risk of misinterpreting survival outcomes due to incomplete data. This preprocessing step resulted in expression data for 39 cancer types along with corresponding overall survival data for the samples. To investigate the relationship between gene expression and survival prognosis in each tumor type, we utilized version 3.2-7 of R package survival to construct a Cox proportional hazards regression model using the coxph function. The Logrank test was used to determine if there were significant differences in prognostic outcomes [15].

### Gene functional enrichment analysis

GeneMANIA (http://genemania.org/) is an online tool for exploring gene interactions and functions and identifying co-expressed genes [17].We used the “Similar Gene Detection “module of GEPIA2 to screen the top 100 DDX17-related target genes.The sangerbox3.0 database was used to obtain the targeted and correlated genes DDX17 pathway, and GO Biological Processes.

### Genetic alteration analysis

The cBioPortal tool (http://www.cbioportal.org/) was utilized to analyze the mutational profile of DDX17 in various cancer types. Additionally, we employed cBioPortal to further investigate the frequency and specific types of mutations occurring in the DDX17 across all tumor samples. The expression data for DDX17 was extracted from the UCSC database, while MuTect2 software was applied to process Simple Nucleotide Variation data obtained from TCGA level samples. To calculate the Tumor Mutational Burden (TMB) for each tumor, we utilized the TMB function within R software’s map tools package and integrated it with both gene expression data and MSI scores derived from a previous study [18].

### DNA and RNA methylation analysis of DDX17

We utilized the UALCAN database to examine the methylation status of DDX17 in both tumor and normal tissues. RNA methylation plays a crucial role as an epigenetic regulatory pathway, contributing to tumor initiation, progression, and prognosis. The types of RNA methylation investigated include 6-methyladenosine (m6A), 5-methylcytidine (m5C), and 1-methyladenosine (m1A). Through Pearson correlation analysis, we investigated the association between DDX17 and regulatory proteins involved in m6A, m5C, and m1A pathways.

### Immune infiltration analysis

We obtained the expression data for the DDX17 gene in each sample from a comprehensive pan-cancer dataset sourced from the UCSC database. Afterwards, we extracted the gene expression profile of each tumor and associated it with GeneSymbol. To assess immunoinfiltration, we utilized ESTIMATE, an R software package, to compute stromal immune, and overall ESTIMATE scores based on gene expression levels for every patient within each tumor. Finally, employing Pearson’s correlation coefficient and utilizing the corr.test function from version 2.1.6 of the psych R package, we identified significant correlations between gene scores and immunoinfiltration scores within individual tumors.

### Immune regulatory gene and immune checkpoints analysis

The association between gene expression and five immune pathway markers (chemokine receptor, MHC, Immunoinhibitor, Immunostimulator) was investigated using the UCSC database, MuTect2 software, and R software [19]. Pearson correlation analysis was performed to assess the relationship between DDX17 gene expression and 60 marker genes from two different types of immune checkpoint pathways (Inhibitory and Stimulatory) [20], which were obtained from the pan-cancer dataset downloaded from the UCSC database for each sample.

### Drug sensitivity analysis

GSCALite(http://bioinfo.life.hust.edu.cn/web/GSCALite/) is an extensive platform designed for the analysis of gene expression and drug sensitivity.

###  Single-Cell functional analysis of DDX17

The CancerSEA (http://biocc.hrbmu.edu.cn/CancerSEA/), serves as an analytical instrument to investigate the functionalities of individual cancer cells. It encompasses a compilation of 14 cellular functions associated with tumors, comprising data from 900 cancer cells across 25 different types of cancers.

### Exploration of DDX17 coexpression networks

LinkedOmics website (http://www.linkedomics.org/login.php) is a visual tool designed to analyze gene expression profiles [19, 21]. By utilizing Pearson’s correlation coefficient, LinkedOmics was employed to identify coexpression genes of DDX17 and the findings were presented through volcano plots and heat maps. Subsequently, an investigation into the Gene Ontology biological process (GO-BP) and DDX17 pathways associated with DDX17 was conducted.

## Results

### Expression levels analysis of DDX17 in pan-cancer

**Fig. 1:**
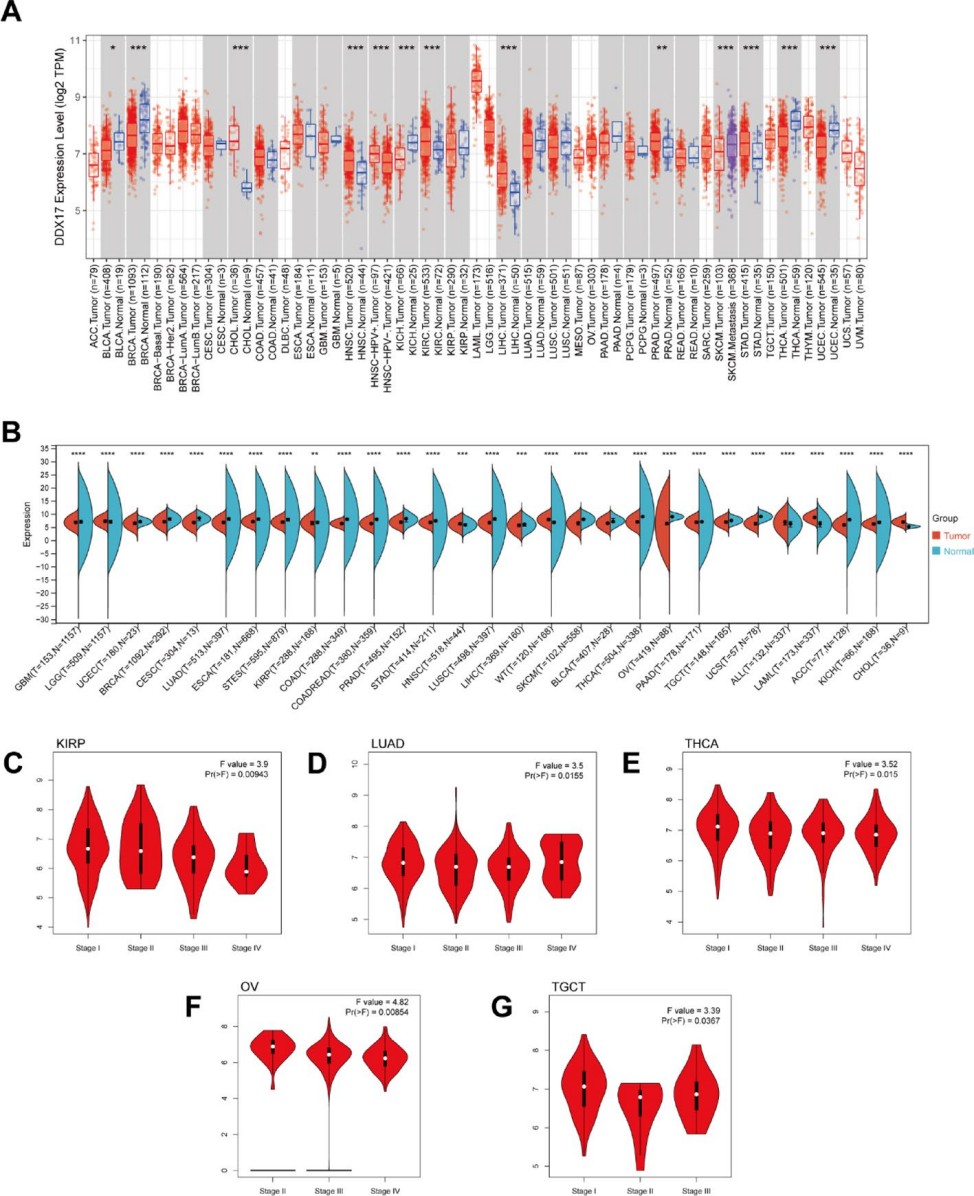
Aberrant expression levels and pathological stages of DDX17 in pan-cancer. **A** The expression status of DDX17 in different tumor types (TCGA database). **B** Combining TCGA and GTEx databases to obtain DDX17 mRNA expression levels. **C**–**G** Correlation between DDX17 expression and pathological stages of KIRP, LUAD, THCA, TGCT, and OV from TCGA datasets. Log2 (TPM + 1) was applied for log-scale. (* *p* < 0.05, ** *p* < 0.01, *** *p* < 0.001)

We conducted an analysis of DDX17 mRNA expression levels in the TCGA database, and observed elevated expression in six cancer types: CHOL, HNSC, KIRC, LIHC, PRAD, and STAD (Fig. 1A). Conversely, reduced expression of DDX17 was noted in four other cancer types: BLCA, KICH, BRCA, THCA, and UCEC. To address the limitation of normal samples in TCGA, we integrated data from the GTEx database with TCGA to assess DDX17 expression across 29 different cancer types. This analysis revealed significant differential expression of DDX17 among all 29 cancer types (Fig. 1B). Utilizing the CPTAC database, we further examined DDX17 protein expression levels and found upregulation in Breast, Colon, Ovarian, Clear cell RCC, UCEC, Lung, PAAD, Head and neck, Glioblastoma, and Liver cancers compared to adjacent normal tissues (*p* < 0.05, Fig S1). We also investigated DDX17 expression across different pathological stages of cancer using the GEPIA2 database. Our analysis shows significant associations (*p* < 0.05, Fig. 1C–G) between DDX17 expression and pathological stages in KIRP, LUAD, THCA, TGCT, and OV. These findings suggest that DDX17 may serve as a potential biomarker for cancer progression and could pave the way for novel targeted therapy research.

### Prognostic value of DDX17 in pan-cancer

**Fig. 2:**
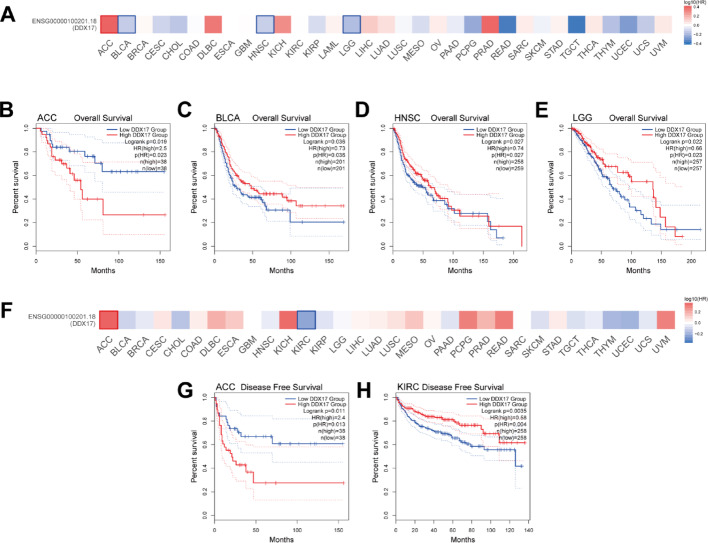
Relationship between DDX17 expression level and prognosis in TCGA tumors. **A** Relationship between DDX17 expression and overall survival. **B**–**E** the positive results of the overall survival map and Kaplan-Meier curves are shown. **F** Relationship between DDX17 expression and disease-free survival. **G**, **H** the positive results of the disease-free survival map and Kaplan-Meier curves are shown

We compared cohorts with high and low DDX17 expression and found that elevated DDX17 expression was associated with improved overall survival (OS) in several tumor types, including BLCA, HNSC, and LGG (Fig. 2A–E). Similarly, the analysis of high versus low DDX17 expression cohorts demonstrated a positive correlation between increased DDX17 expression and better disease-free survival (DFS) in KIRC (Fig. 2F–H). These findings demonstrate that elevated DDX17 expression is significantly associated with improved prognosis, suggesting that patients with high DDX17 levels may exhibit reduced recurrence rates and enhanced survival outcomes. Collectively, these results highlight DDX17 as a promising prognostic biomarker in cancer.

### Functional enrichment analysis of DDX17

**Fig. 3:**
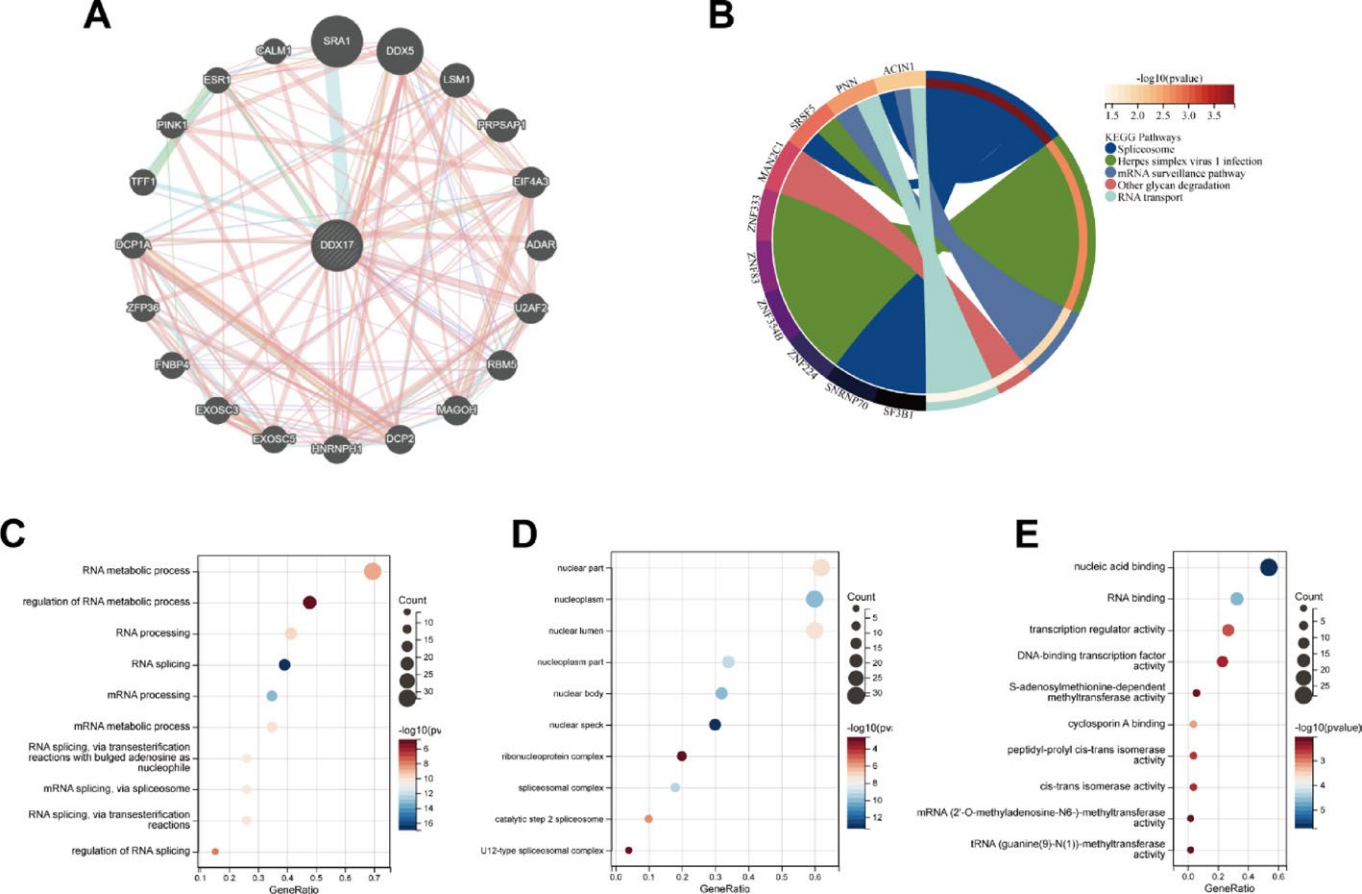
DDX17-related gene enrichment analysis. **A** Twenty genes co-expressed with DDX17 in GeneMANIA. **B** DDX17 pathway analysis based on the DDX17-interacted and correlated genes. **C** GO-BP enrichment analysis of DDX17 related genes. **D** GO-CC enrichment analysis of DDX17 related genes. **E** GO-MF enrichment analysis of DDX17 related genes

To investigate the potential role of DDX17 in tumorigenesis, we extracted 20 genes associated with DDX17 from the GeneMANIA database (Fig. 3A). Additionally, we identified 100 DDX17-related genes from the TCGA database using GEPIA2. Subsequently, we conducted an enrichment analysis for DDX17, which revealed associations with five signaling pathways: Spliceosome, Herpes simplex virus 1 infection, mRNA surveillance pathway, Other glycan degradation, and RNA transport (Fig. 3B). GO analysis further implicated DDX17 in RNA metabolic processes, regulation of RNA metabolic processes, RNA processing, RNA splicing, and other biological processes (Fig. 3C). The cellular component (CC) analysis of DDX17-related genes predominantly localized to the nuclear part, nuclear body, and nucleoplasm (Fig. 3D). Molecular function (MF) analysis indicated that DDX17 is involved in nucleic acid binding, RNA binding, transcriptional regulation, DNA-binding transcription factor activity, and other related processes (Fig. 3E).These pathways are critical for maintaining cellular homeostasis and are often dysregulated in cancer. For instance, aberrant RNA splicing can lead to the production of oncogenic isoforms, promoting tumor progression [10]. The involvement of DDX17 in these processes suggests that it may play a central role in regulating cancer-related RNA metabolism, making it a potential therapeutic target for cancers with dysregulated RNA processing.

### DDX17 expression and immune infiltration in Pancancer research

**Fig. 4:**
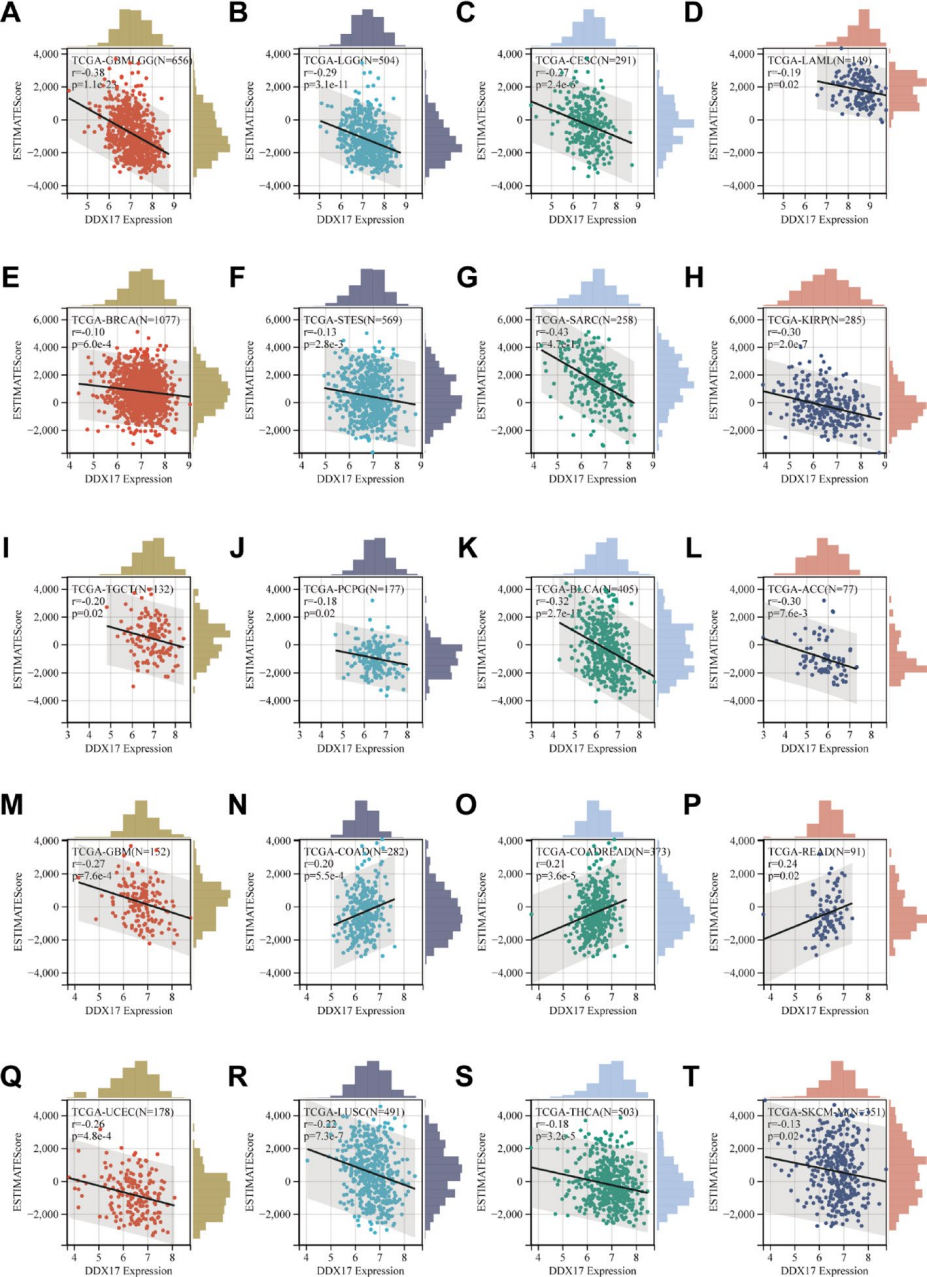
Immune infiltration analysis of DDX17 in different tumors. Analysis of DDX17 expression and immune infiltration score in tumor types (**A**–**T**)

We investigated the impact of DDX17 on tumor immunomodulation by analyzing the correlation between DDX17 expression and immune infiltration in tumors. Our results demonstrated a significant association(*p* < 0.05) between DDX17 expression levels and immune infiltration scores in tumors. Notably, a positive correlation was observed between DDX17 expression and immune infiltration in three specific cancer types: COAD, COADREAD, and READ, suggesting that DDX17 may promote immune cell recruitment in these cancers. Conversely, a significant negative correlation was found between DDX17 expression and immune infiltration in seventeen other cancer types, including GBM, GBMLGG, LGG, CESC, LAML, BRCA, STES, SARC, KIRP, UCEC, LUSC, THCA, SKCM-M, TGCT, PCPG, ACC, and BLCA (Fig. 4A–T), implying that DDX17 might suppress immune infiltration, potentially contributing to immune evasion in these tumors.

### DDX17 expression and immune regulatory gene, immune checkpoints and drug sensitivity

**Fig. 5:**
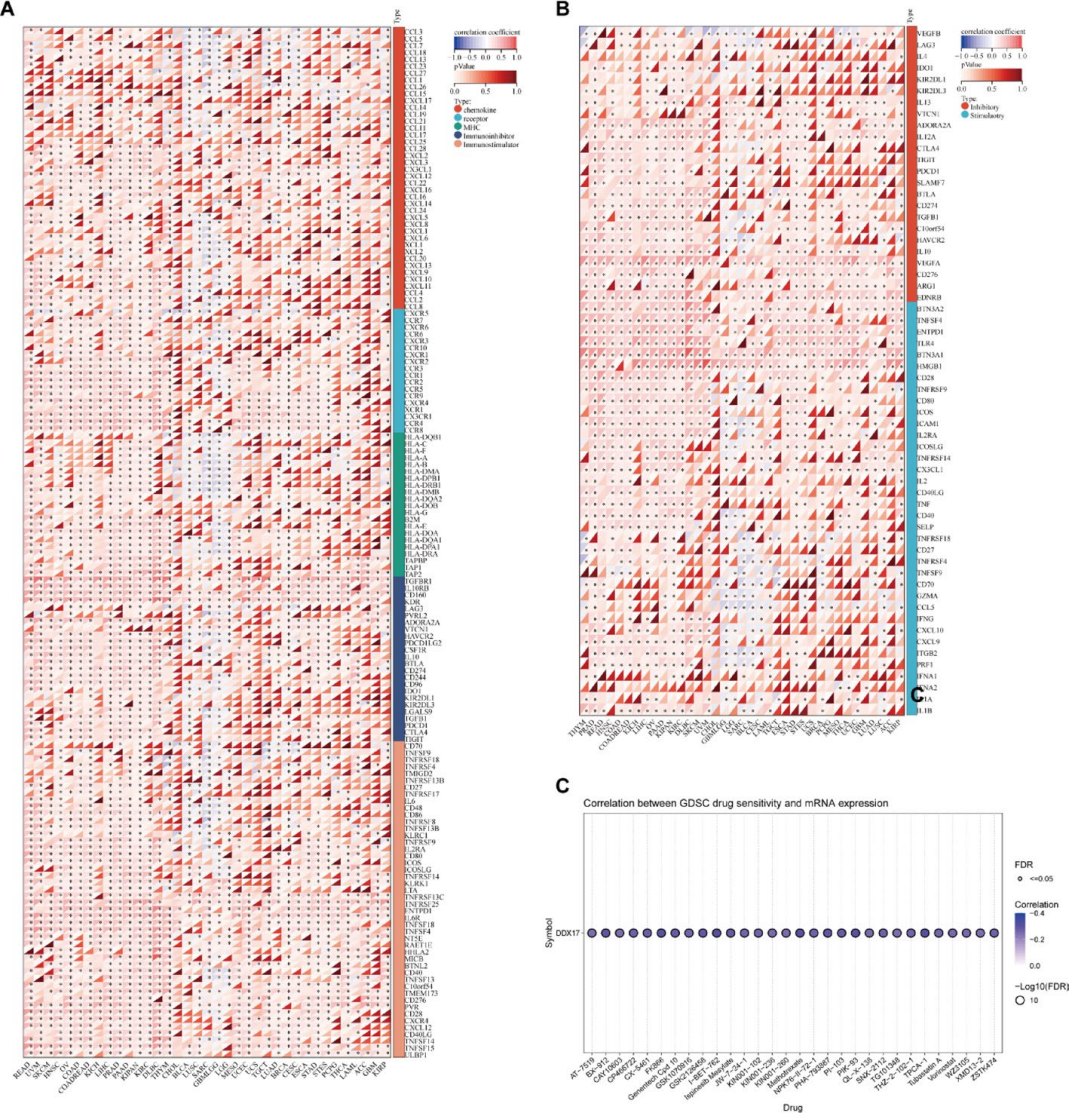
Correlation analysis of DDX17 and immune regulatory gene, immune checkpoints and drug sensitivity. **A** The correlation of DDX17 expression with most immune regulatory gene. **B** The correlation of DDX17 and known immune checkpoints across all TCGA cancers. **C** The associations of DDX17 expression and drug sensitivity base on GSCALite. (* *p* < 0.05)

We subsequently analyzed the relationship between DDX17 levels and immune-modulating genes, immune checkpoints, and drug sensitivity. Initially, we utilized a pan-cancer dataset from the UCSE database for our study. We extracted DDX17 and 150 marker genes, carefully filtering the expression data of these markers in each sample. We then calculated the Pearson correlation coefficients between DDX17 and five immune pathway marker genes. Notably, a positive correlation was observed between DDX17 expression and the majority of immunomodulatory genes (Fig. 5A). We further examined the relationship between DDX17 and specific immune checkpoints. Based on the data, a significant correlation was observed between DDX17 and the majority of immune checkpoints (Fig. 5B). Additionally, we used GSCALite to explore the impact of DDX17 expression on drug sensitivity in tumors. We found an inverse association between DDX17 expression and the 50% inhibitory concentration (IC50) values for all drugs tested (Fig. 5C).

### Mutation analysis of DDX17 in the TCGA pan-cancer cohort

**Fig. 6:**
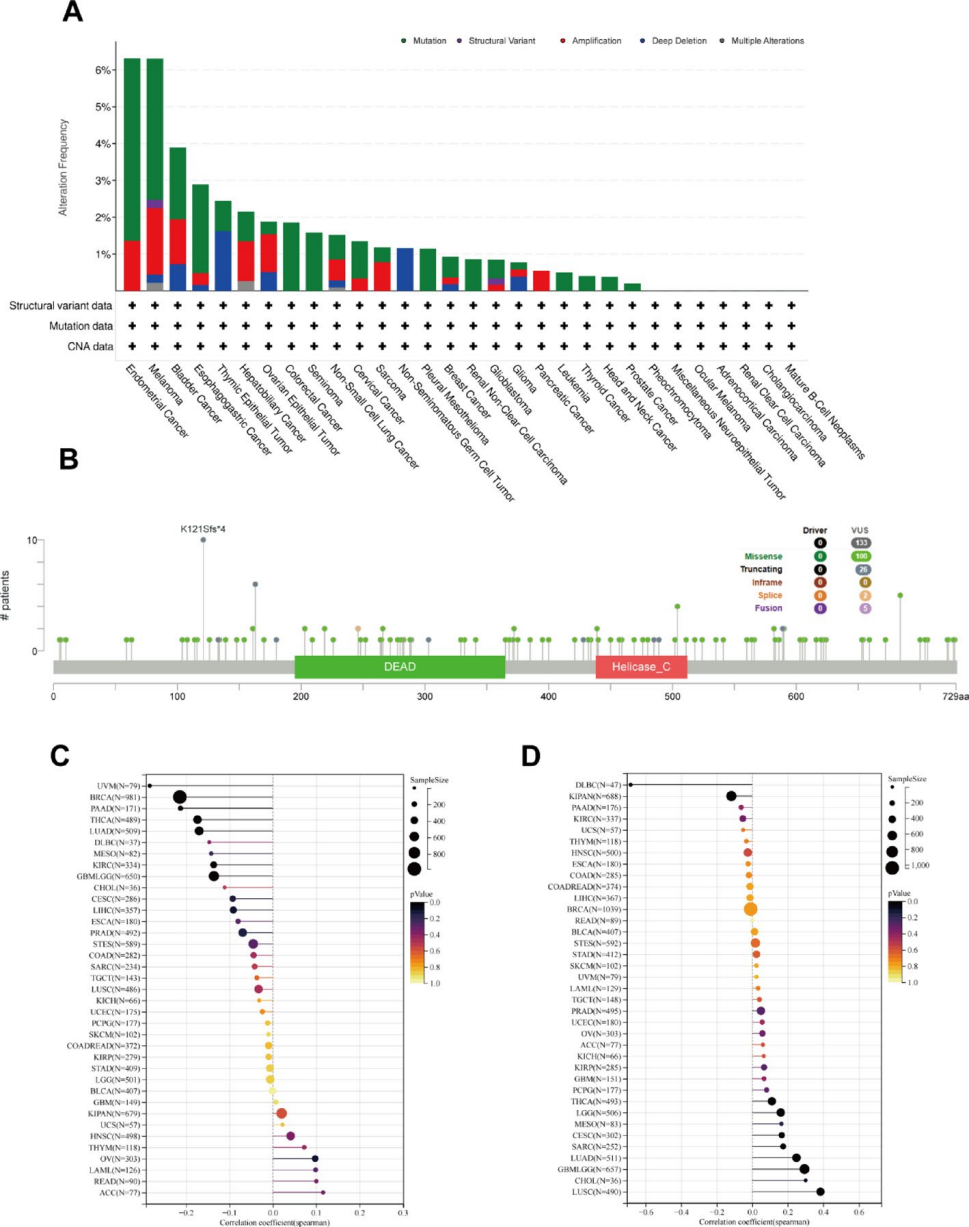
**A** Genetic alterations of DDX17 in pan-cancer using the cBioPortal. **B** The mutation site of DDX17 and the number of related cases at this mutation site. The association between DDX17 among TMB levels (**C**) and MSI (**D**)

To elucidate the potential role of DDX17 in cancer development via genetic alterations, we analyzed the specific sites and types of mutations within the DDX17, along with RNA modifications and genomic heterogeneity across various cancer types. Among the 32 cancer types analyzed, endometrial cancer had the highest frequency of DDX17 alterations, at 6.80% (Fig. 6A). Our results shed light on the specific types, locations, and frequencies of DDX17 gene mutations. Notably, missense mutations were the most prevalent form of genetic alteration in DDX17 (Fig. 6B and Table S2). Furthermore, we explored the relationship between DDX17 expression and TMB (tumor mutational burden), both of which are emerging biomarkers for immunotherapy response. Analysis of data from 37 tumors showed a significant negative correlation between TMB and DDX17 expression in seven cancer types (LUAD, GBMLGG, BRCA, KIRC, THCA, PAAD, UVM) (Fig. 6C). Additionally, our analysis revealed associations between DDX17 expression and MSI (microsatellite instability) across different cancers, with positive correlations observed for GBMLGG, LGG, CESC, LUAD, SARC, LUSC, and THCA, while negative correlations were found for KIPAN and DLBC (Fig. 6D).

### The association between DDX17 expression and DNA methylation and RNA methylation

**Fig. 7:**
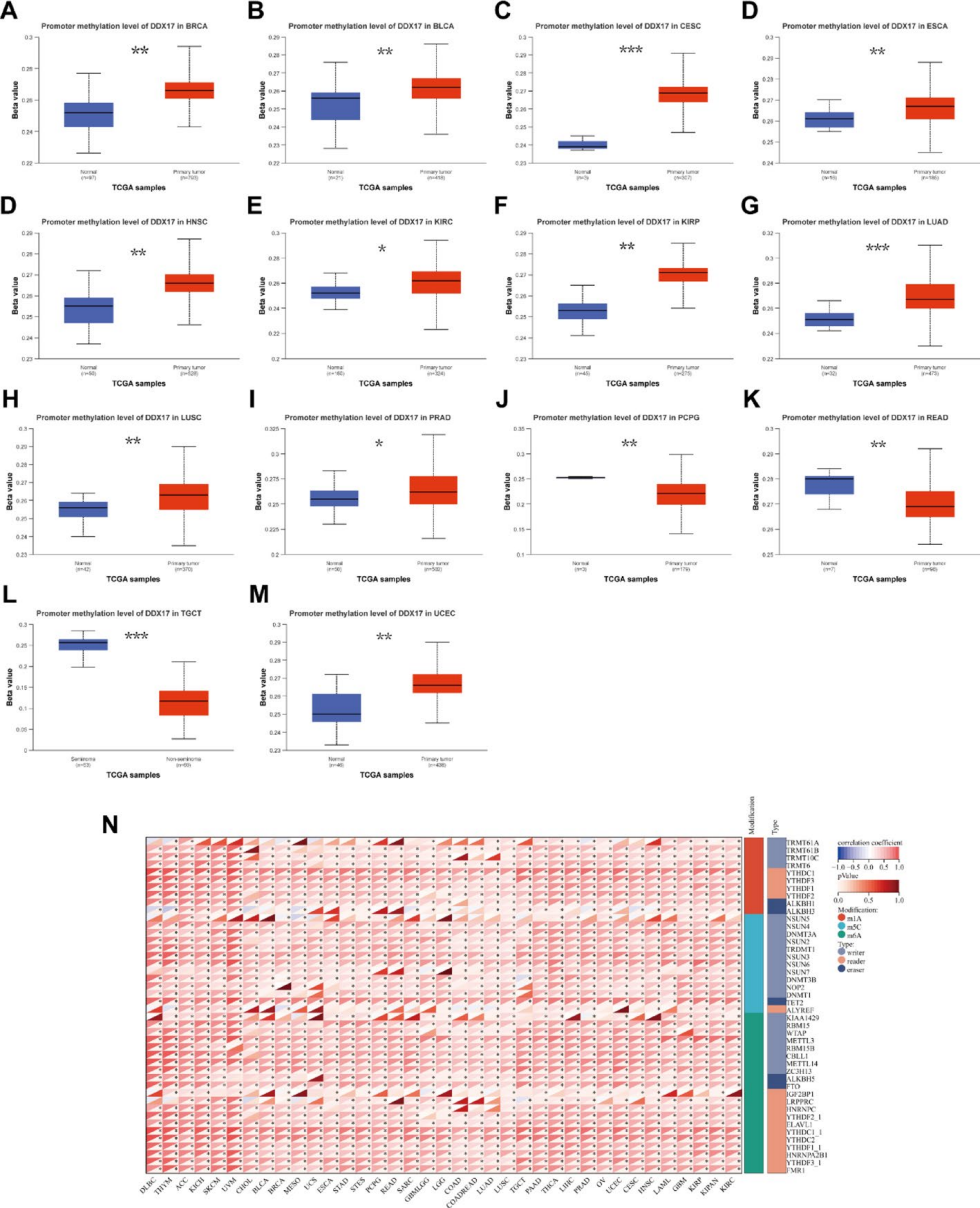
Relationship of DDX17 with methylation and methyltransferase. **A**–**M** Promoter methylation level of DDX17 in various tumors. **N** The correlation between DDX17 expression and m1A, m5C, m6A regulatory genes. (* *p* < 0.05, ** *p* < 0.01, *** *p* < 0.001)

We investigated the relationship between DDX17 expression and both DNA and RNA methylation. Using data from the UALCAN database, we assessed the DNA methylation levels of DDX17 across various tumor types. Our results indicated significantly higher methylation levels of DDX17 in most tumor types compared to normal tissues, which may contribute to the increased expression of DDX17 in these tumors (Fig. 7A-M). Additionally, we observed positive correlations between DDX17 and key regulatory genes associated with m6A, m5C, and m1A modifications in multiple cancer types. These findings suggest that changes in the expression of RNA methylation regulatory genes could play a role in the carcinogenic potential of DDX17 (Fig. 7N).

### Single-cell functional analysis of DDX17

**Fig. 8:**
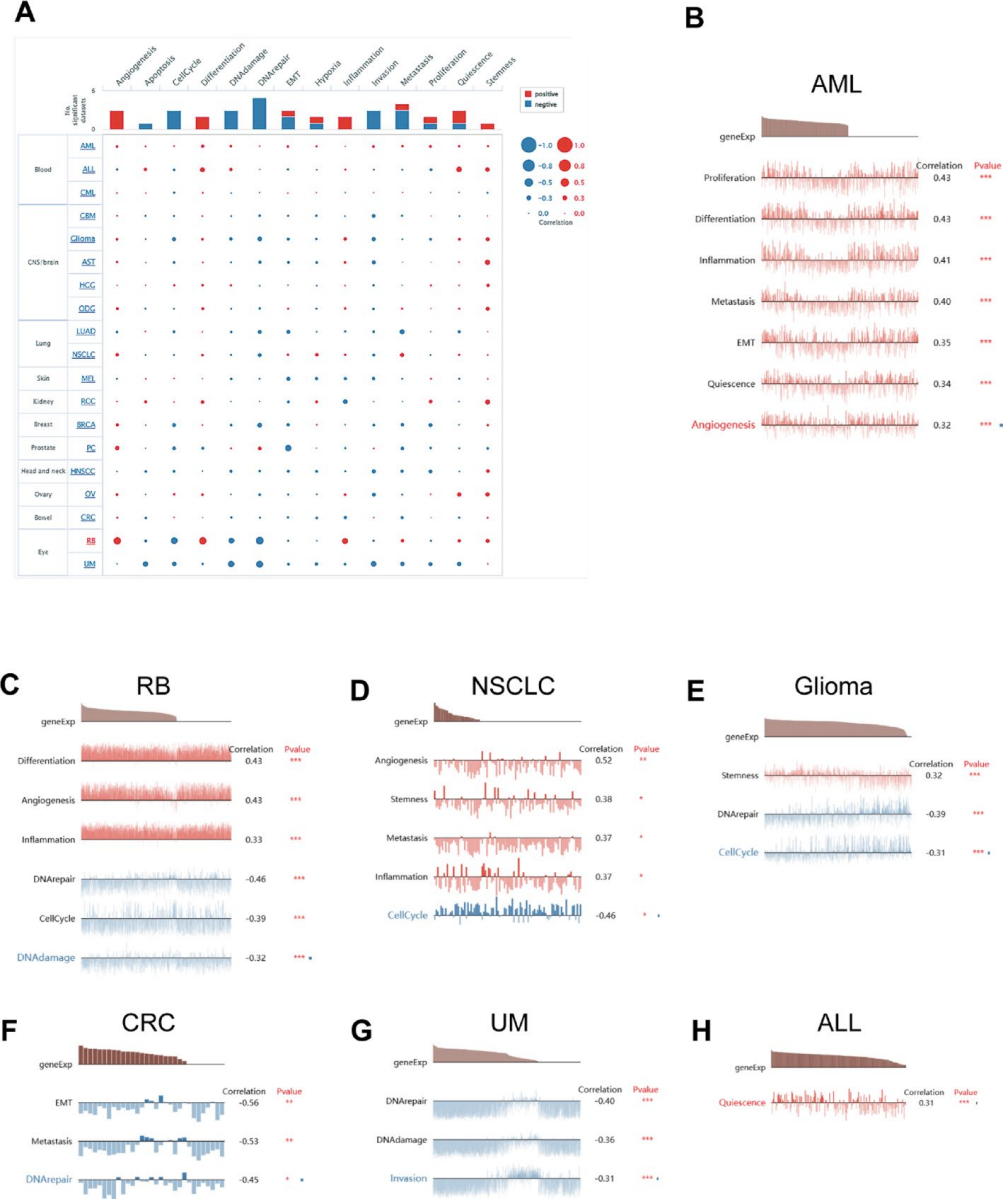
The function of DDX17 in single-cell functional analysis from the CancerSEA database. **A** Functional status of DDX17 in different human cancers. **B**–**H** Correlation analysis between functional status and DDX17 in AML, RB, NSCLC, Glioma, CRC, UM, and ALL. (* *p* < 0.05, ** *p* < 0.01, *** *p* < 0.001)

To elucidate the intrinsic role of DDX17 in tumorigenesis, we performed a comprehensive cell-level analysis of its functions using CancerSEA (Fig. 8A). Our results highlighted a robust positive correlation between DDX17 and critical biological processes in AML, including proliferation, differentiation, inflammation, metastasis, epithelial-mesenchymal transition (EMT), quiescence, and angiogenesis (Fig. 8B). In retinoblastoma (RB), DDX17 was positively correlated with differentiation, angiogenesis, and inflammation, but negatively correlated with DNA repair, cell cycle, and DNA damage (Fig. 8C). In non-small cell lung cancer (NSCLC), DDX17 showed significant positive correlations with angiogenesis, stemness, metastasis potential, and inflammatory responses, while it negatively correlated with the cell cycle (Fig. 8D). In glioma, DDX17 expression was positively associated with stemness and negatively correlated with DNA repair and cell cycle regulation (Fig. 8E). In contrast, in colorectal cancer (CRC), DDX17 levels exhibited a negative correlation with EMT, which is implicated in tumor invasion, migration, and metastasis, as well as DNA repair (Fig. 8F). Similarly, in uveal melanoma (UM), DDX17 expression was unfavorably associated with DNA damage, invasion potential, and DNA repair efficiency (Fig. 8G). Conversely, in acute lymphoblastic leukemia (ALL), a positive correlation was observed between DDX17 expression and quiescence (Fig. 8H).

### TLIHC patients: the function analysis of DDX17 in LIHC

**Fig. 9:**
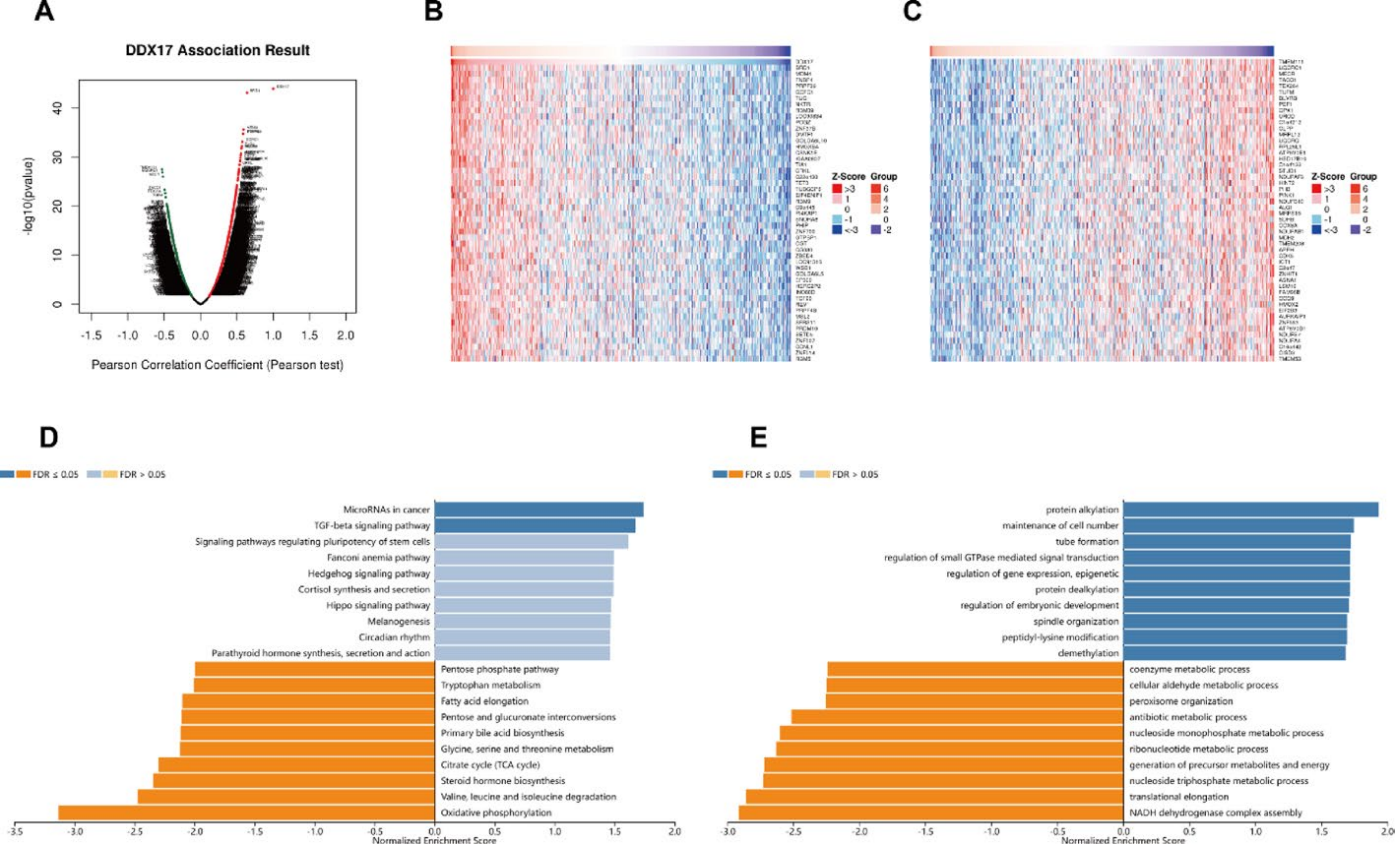
Function and pathway enrichment analyses for genes co-expressed with DDX17 in LIHC. **A** Highly correlated genes of DDX17 tested by Pearson test in LIHC cohort; Top 50 positive coexpression genes (**B**) and negative coexpression genes (**C**) of DDX17 in heat map in LIHC (**D**) DDX17 analysis of DDX17 co-expression genes in the LIHC cohort (**E**) GO analysis of DDX17 co-expression genes in the LIHC cohort

Based on the aforementioned findings, DDX17 is significantly associated with cancer immunity and prognosis. We subsequently used the LinkedOmics database to explore the coexpression networks of DDX17, thereby confirming its functional significance in tumor tissues. Specifically, our analysis in liver hepatocellular carcinoma (LIHC) revealed that genes represented by dark red dots were positively correlated with DDX17, whereas those by dark green dots were negatively correlated (Fig. 9A). The heatmaps display the top 50 genes that are positively and negatively associated with DDX17, respectively (Fig. 9B, C). KEGG analysis indicated enrichment in pathways such as MicroRNAs in cancer and the TGF-beta signaling pathway (Fig. 9D). Additionally, GO term annotation revealed that co-expressed genes of DDX17 are primarily involved in processes like protein alkylation, cell number maintenance, tube formation, and regulation of small GTPase-mediated signal transduction (Fig. 9E). These results suggest that the expression network of DDX17 influences prognosis and immune activation in LIHC.

## Discussion

The global incidence of cancer-related deaths is rapidly increasing, making it the leading cause of mortality worldwide [22]. Among all types of cancer, female breast carcinoma has the highest prevalence with approximately 2.3 million new cases reported annually (11.7%). The most common cancers include lung (11.4%), colorectal (10.0%), prostate (7.3%), and stomach (5.6%) [23]. Lung, colorectal, liver, stomach, and breast cancer among females exhibit the most elevated fatality rates [23]. Surgery, radiation therapy, and additional chemotherapy are frequently employed in the treatment of cancer; however, their efficacy is somewhat restricted [24]. The selection of cancer therapy should be determined by the unique circumstances surrounding each patient. Extensive research on various types of cancer can enhance our comprehension of shared traits and mechanisms across different forms of the disease. Such investigations contribute to the identification of disease mechanisms, biomarkers, and treatment options [25].

DDX17 is gaining significant attention for its important roles in multiple pathophysiological processes [8]. DDX17 influences cancer initiation and progression through a variety of mechanisms, such as transcriptional regulation, RNA binding, and formation of the microprocessor complex as a member of the Kinesin family, is a crucial regulating factor of chromosome arrangement during mitosis [26]. Recent studies have shown that DDX17 participates in the pathogenesis and progression of various tumors, such as colorectal cancer, liver cancer, pancreatic ductal adenocarcinoma, and lung adenocarcinoma, and is also related to these tumors’ survival and prognosis [9, 27–29]. We used a variety of databases from TCGA, GTEx, UALCAN, cBioportal, and others to reveal the molecular characteristics of DDX17 in 33 tumors from an overall perspective, including gene expression, prognosis, gene alterations, immune infiltration, DNA methylation, RNA methylation, and drug sensitivity to clarify its role in the development and potential regulatory pathways of different tumor.In this study, integrating data from multiple sources presented several challenges, including differences in data formats, normalization methods, and platform-specific biases. To address these challenges, we harmonized the data by using common identifiers, standardizing preprocessing steps, and applying cross-platform normalization techniques. Additionally, we used the Sangerbox online tool to ensure consistency in data processing across different datasets. These steps ensured the comparability and reliability of our integrated analysis.

In this study, we discovered the significant overexpression of DDX17 in 27 cancers from TCGA and GTEx databases. The DDX17 was substantially expressed in the majority of malignancies and was related to pathological staging in cancers such as KIRP, LUAD, THCA, TGCT, and OV. Subsequent studies have revealed that higher DDX17 expression is associated with improved overall and disease-free survival in specific tumor types, including BLCA, HNSC, and LGG. This indicates that DDX17 could function as a promising prognostic indicator, enabling us to forecast patient survival and disease progression. This breakthrough could facilitate the creation of therapeutic methods that focus on DDX17, underscoring the importance of DDX17 immunotherapy in oncology.

Enrichment analysis of DDX17 implicated its involvement in several signaling pathways, including the Spliceosome, Herpes simplex virus 1 infection, mRNA surveillance pathway, glycan degradation, and RNA transport. Gene Ontology (GO) enrichment analysis further revealed that DDX17 is likely engaged in RNA metabolism, including the regulation of RNA metabolic processes, RNA processing, and RNA splicing. These findings align with those of previous studies, which have shown that DDX17 regulates alternative splicing, leading to the production of an oncogenic isoform of PXN-AS1 and promoting hepatocellular carcinoma (HCC) metastasis. Additionally, DDX17 modulates the expression and alternative splicing of genes involved in apoptosis and proliferation in lung adenocarcinoma cells.

The tumor microenvironment encompasses the nurturing conditions for both the tumor and infiltrating immune and stromal cells [30]. A growing body of research evidence underscores the pivotal role of immune cell infiltration in tumorigenesis and the efficacy of immunotherapy. The positive correlation between DDX17 and immune infiltration in COAD and READ suggests that DDX17 may enhance anti-tumor immunity in these cancers, making it a potential target for immunotherapy. Conversely, the negative correlation in GBM、LGG and other cancer types implies that DDX17 might contribute to immune evasion, suggesting that targeting DDX17 could improve the efficacy of immune checkpoint inhibitors in these cancers. Recent studies suggest that immune scores may serve as a valuable biomarker for evaluating cancer patients’ survival outcomes, recurrence risks, metastatic potential, and drug resistance [31]. Our analysis revealed a positive association between DDX17 expression and both stromal and immune scores in three distinct types of cancer. In contrast, a negative association was identified in nine additional types. These results imply that DDX17 contributes to the complex interplay between tumor and immune cells, potentially influencing tumor microenvironments. Consequently, these insights may pave the way for the development of more targeted and effective therapeutic strategies.

Immune checkpoint genes serve as crucial targets for immune checkpoint inhibitors (ICIs) when treating diverse forms of cancer [32]. The current state of affairs indicates that ICIs serve as a highly efficacious immunotherapeutic modality for combating cancer [33]. Furthermore, DDX17 expression exhibited a robust positive correlation with multiple immune checkpoints typically present in tumors, indicating that DDX17 may serve as a novel target for immunotherapeutic strategies against cancer. Our study also identified significant interactions between DDX17 and genes implicated in immune regulation. These results underscore the tight link between DDX17 expression and immune cell infiltration within tumors, which could significantly affect patient prognosis and identify new targets for immunosuppressive therapy development. Additionally, we investigated the relationship between DDX17 mRNA levels and the sensitivity of anti-cancer drugs. We found that elevated DDX17 expression was negatively correlated with IC50 values for 30 distinct types of anti-cancer drugs, as per data from the GDSC database. This correlation suggests that these drugs may effectively halt cancer progression, offering critical guidance for the clinical selection of drugs and the prediction of patient responses.

Given the diminished expression of DDX17 in certain tumors relative to normal tissues, an in-depth analysis of DDX17 mutations was conducted, drawing on previous literature and data from the TCGA database. This study revealed that mutations in DDX17 are prevalent across the majority of tumors, suggesting a pivotal role in tumorigenesis. Tumor mutational burden (TMB) and microsatellite instability (MSI) are established biomarkers for prognostic evaluation in various cancers and predictors of immunotherapeutic response in numerous tumors [34, 35]. Tumors with high TMB and high MSI, have better response to immunotherapy [36]. Our research findings demonstrate a significant inverse association between the expression of DDX17 and tumor mutational burden (TMB) across seven distinct types of tumors. Furthermore, we observed a negative correlation between DDX17 expression and microsatellite instability (MSI) in KIPAN and DLBC. These results suggest that patients diagnosed with these nine tumors who exhibit low levels of DDX17 expression may potentially derive enhanced outcomes through the utilization of immunotherapy.

DNA methylation is a commonly occurring epigenetic modification that plays a crucial role in the regulation of gene expression, maintenance of genomic stability, and promotion of tumor formation. Previous studies have shown that abnormal DNA methylation can accelerate tumor development by influencing cell proliferation, leading to apoptosis or senescence [37]. By utilizing the UALCAN tool, we observed a significant rise in DDX17 promoter methylation levels across most tumors compared to normal tissues, except for PCPG and READ where it decreased. RNA methylation has been implicated in tumorigenesis, development, and prognosis. In our study, we discovered a positive association between DDX17 expression and regulatory genes involved in RNA methylation for various cancers. These findings suggest that DDX17 may contribute to tumorigenesis through its involvement in RNA methylation.We used CancerSEA and GSEA to perform pan-cancer functional analyses of DDX17. Single-cell function analysis showed that DDX17 was positively related to cell cycle and proliferation in some tumors. Recent studies have shown that the inhibition of DDX17 expression in HCC could reduce cell cycle-related protein expression levels, and DDX17 might promote HCC cell proliferation through the cell cycle signaling pathway.GSEA analysis of DDX17 revealed that DDX17 was primarily involved in the DNArepair, DNA damage, angiogenesis, stemness, metastasis, and inflammation of some cancers. This is consistent with previous research, DDX17 can promotes efficient repair of DNA double-strand breaks (DSBs) at genomic regions with low propensity for DNA: RNA-hybrid formation by facilitating the generation of DSB-induced hybrids, thus enabling effective propagation of the cellular response to DNA damage [38], microprocessor complex regulates the cell cycle progression by degrading Aurora kinase B mRNA, and this process is regulated by RNA helicases DDX5 and DDX17 [39]. However, the interaction between DDX17 and the angiogenesis is not completely comprehended and needs to be investigated further in future work.

Overall, this extensive examination of cancer highlights the importance of DDX17 in prognostic assessment and cancer classification, particularly in relation to immunotherapy. Further exploration into the underlying mechanisms of DDX17 is likely to enhance our comprehension of its role in tumor biology and offer innovative therapeutic options for individuals with cancer. The discoveries from this study have the potential to positively influence future clinical procedures, leading to improved outcomes for patients with cancer. However, it is crucial to acknowledge certain limitations despite describing the impact of DDX17 across various types of cancer. Although we analyzed DDX17 through a comprehensive and systematic process, using multiple databases and R 4.1.0 to cross-verify, some limitations remain. Initially, the inconsistency in sequencing and microarray data across various databases, coupled with a lack of specificity and granularity, may introduce systematic bias into our analysis. Therefore, conducting in vivo or in vitro experiments to ascertain the functional role of DDX17 is essential for enhancing the reliability of our results. Additionally, Our study did not delve into the molecular mechanisms underlying the role of DDX17 in cancers, leaving this area ripe for future investigation. Future research should focus on clarifying the regulatory mechanisms of DDX17 expression in tumors, and further research is warranted to understand its involvement in the immune response within the tumor microenvironment.

## Conclusion

Our study systematically investigated the significant correlation between DDX17 expression and clinical characteristics, prognosis, mutational status, DNA methylation, RNA methylation, TMB, TMB, MSI, and drug sensitivity across various cancer types. This comprehensive analysis enhances our understanding of the potential involvement of DDX17 in pan-cancer.

## Limitations

Although we analyzed DDX17 through a comprehensive and systematic process, using multiple databases and R language to cross-verify, some limitations remain. Initially, the inconsistency in sequencing and microarray data across various databases, coupled with a lack of specificity and granularity, may introduce systematic bias into our analysis. Therefore, conducting in vivo or in vitro experiments to ascertain the functional role of DDX17 is essential for enhancing the reliability of our results. Additionally, Our study did not delve into the molecular mechanisms underlying the role of DDX17 in cancers, leaving this area ripe for future investigation.

## Electronic supplementary material

Below is the link to the electronic supplementary material.

Supplementary Material 1

## Data Availability

All data needed to evaluate the conclusions in the paper are present in the paper and the Supplementary Materials.
