# Supplementary material for: Prognostic and immunological landscape of DDX17 in pan-cancer analysis: a comprehensive study
Source: Discov Oncol. 2025 Jun 17;16:1132. doi: 10.1007/s12672-025-02955-9 (PMC12173986; doi:10.1007/s12672-025-02955-9)
Supplement: Supplementary file 1 — Supplementary Material 1 [file 12672_2025_2955_MOESM1_ESM.docx]

**Supporting information**

**Prognostic and Immunological Landscape of DDX17 in Pan-Cancer Analysis: A Comprehensive Study**

Yu Lei^1a^, Xin He^1b^, Peng Chen^1a^ , Yiping Qin^c^, Bin Ge^a^, Yan Liu^a^, Pu Li^b*^, Xing Wei^a*^

^a^ Department of Clinical Laboratory, Pidu District People's Hospital, Third Affiliated Hospital of Chengdu Medical College, Chengdu , Sichuan, China. 611730, P.R. China

^b^ Department of Clinical Laboratory, Chongqing University Jiangjin Hospital, School of Medicine, Chongqing University, Jiangjin, Chongqing, China. 402260, P.R. China

^c^ Chongqing Key Laboratory of Translational Research for Cancer Metastasis and Individualized Treatment, Chongqing University Cancer Hospital, Chongqing 400030, China

^1^ Parallel first author

*Correspondence authors:

Xing Wei, Department of Clinical Laboratory, Pidu District People's Hospital, Third Affiliated Hospital of Chengdu Medical College, Sichuan, China. 611730, P.R. China.

E-mail address: weixing3009@163.com

Pu Li, Department of Clinical Laboratory, Chongqing University Jiangjin Hospital, School of Medicine, Chongqing University, Jiangjin, Chongqing, China. 402260, P.R. China.

E-mail address: lipu.cqu@cqu.edu.cn.

Fax.: 86-23-47521342


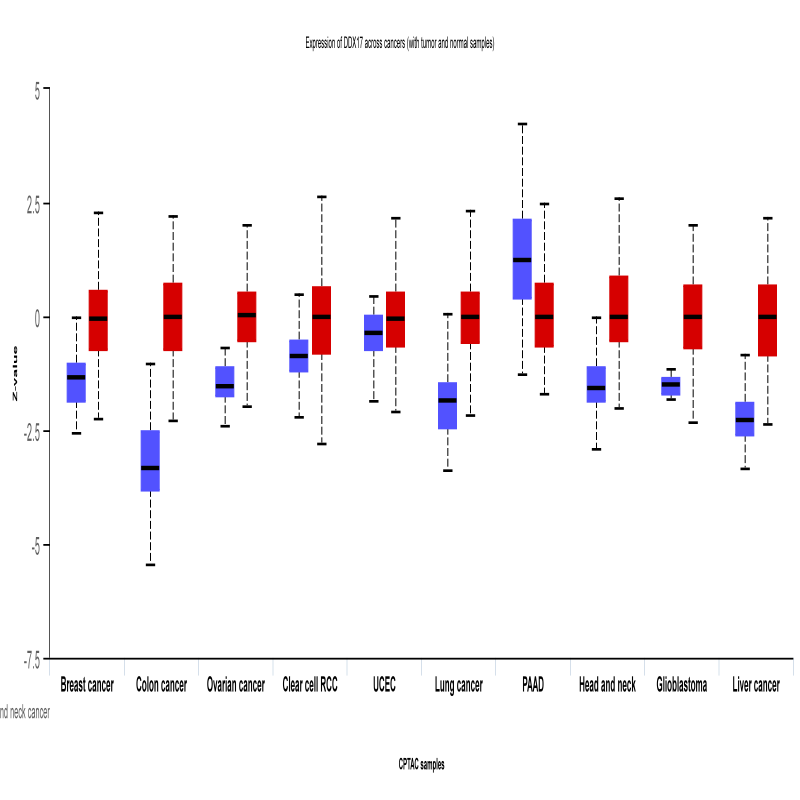


**Figure S1:** DDX17 total protein levels between cancer tissues and normal tissues were shown by CPTAC and the red module represents the cancer tissue.

**Table S1**. Abbreviations from the TCGA database for 33 cancers.

| **Cancer Type** | **Abbreviation** |
| --- | --- |
| **ACC** | Adrenocortical carcinoma |
| **BLCA** | Bladder Urothelial Carcinoma |
| **BRCA** | Breast invasive carcinoma |
| **CESC** | Cervical squamous cell carcinoma and endocervical adenocarcinoma |
| **CHOL** | Cholangiocarcinoma |
| **COAD** | Colon adenocarcinoma |
| **DLBC** | Lymphoid Neoplasm Diffuse Large B-cell Lymphoma |
| **ESCA** | Esophageal carcinoma |
| **GBM** | Glioblastoma multiforme |
| **HNSC** | Head and Neck squamous cell carcinoma |
| **KICH** | Kidney Chromophobe |
| **KIRC** | Kidney renal clear cell carcinoma |
| **KIRP** | Kidney renal papillary cell carcinoma |
| **LAML** | Acute Myeloid Leukemia |
| **LGG** | Brain Lower Grade Glioma |
| **LIHC** | Liver hepatocellular carcinoma |
| **LUAD** | Lung adenocarcinoma |
| **LUSC** | Lung squamous cell carcinoma |
| **MESO** | Mesothelioma |
| **OV** | Ovarian serous cystadenocarcinoma |
| **PAAD** | Pancreatic adenocarcinoma |
| **PCPG** | Pheochromocytoma and Paraganglioma |
| **PRAD** | Prostate adenocarcinoma |
| **READ** | Rectum adenocarcinoma |
| **SARC** | Sarcoma |
| **STAD** | Stomach adenocarcinoma |
| **SKCM** | Skin Cutaneous Melanoma |
| **TGCT** | Testicular Germ Cell Tumors |
| **THCA** | Thyroid carcinoma |
| **THYM** | Thymoma |
| **UCEC** | Uterine Corpus Endometrial Carcinoma |
| **UCS** | Uterine Carcinosarcoma |
| **UVM** | Uveal Melanoma |

**Table S2**. Mutation Analysis of DDX17 in the TCGA pan-cancer cohort.

| **Cancer Type** | **Sample ID** | **Protein Change** | **Mutation Type** |
| --- | --- | --- | --- |
| Glioblastoma | TCGA-06-2565-01 | CSNK1E-DDX17 Fusion | fusion |
| Non-Small Cell Lung Cancer | TCGA-50-6594-01 | ASPHD2-DDX17 Fusion | fusion |
| Hepatobiliary Cancer | TCGA-KR-A7K8-01 | DDX17-ENTHD1 Fusion | fusion |
| Melanoma | TCGA-D3-A1Q3-06 | DDX17-CDH10 Fusion | fusion |
| Melanoma | TCGA-EE-A2MU-06 | DDX17-CPT1B Fusion | fusion |
| Thyroid Cancer | TCGA-DJ-A2QB-01 | R480S | Missense_Mutation |
| Thyroid Cancer | TCGA-FE-A23A-01 | P63Q | Missense_Mutation |
| Glioma | TCGA-DU-6410-01 | R329G | Missense_Mutation |
| Ovarian Epithelial Tumor | TCGA-25-1326-01 | R589* | Nonsense_Mutation |
| Ovarian Epithelial Tumor | TCGA-04-1651-01 | R161S | Missense_Mutation |
| Glioblastoma | TCGA-19-2629-01 | K428Efs*4 | Frame_Shift_Del |
| Glioblastoma | TCGA-28-5207-01 | K535R | Missense_Mutation |
| Glioblastoma | TCGA-06-5416-01 | L226F | Missense_Mutation |
| Glioblastoma | TCGA-06-5416-01 | S219P | Missense_Mutation |
| Glioblastoma | TCGA-06-5416-01 | R154Q | Missense_Mutation |
| Sarcoma | TCGA-DX-A8BK-01 | R209W | Missense_Mutation |
| Non-Small Cell Lung Cancer | TCGA-22-5478-01 | R684G | Missense_Mutation |
| Non-Small Cell Lung Cancer | TCGA-85-8350-01 | R586Q | Missense_Mutation |
| Bladder Cancer | TCGA-2F-A9KT-01 | D332H | Missense_Mutation |
| Bladder Cancer | TCGA-4Z-AA7Q-01 | D395G | Missense_Mutation |
| Bladder Cancer | TCGA-G2-AA3D-01 | E126K | Missense_Mutation |
| Bladder Cancer | TCGA-XF-AAML-01 | L248I | Missense_Mutation |
| Bladder Cancer | TCGA-XF-AAML-01 | P252S | Missense_Mutation |
| Bladder Cancer | TCGA-DK-AA75-01 | S400T | Missense_Mutation |
| Bladder Cancer | TCGA-E5-A4TZ-01 | R684G | Missense_Mutation |
| Bladder Cancer | TCGA-4Z-AA84-01 | S672L | Missense_Mutation |
| Bladder Cancer | TCGA-XF-AAML-01 | E583K | Missense_Mutation |
| Bladder Cancer | TCGA-YC-A89H-01 | G590E | Missense_Mutation |
| Leukemia | TCGA-AB-2926-03 | X246_splice | Splice_Site |
| Hepatobiliary Cancer | TCGA-XR-A8TF-01 | A469E | Missense_Mutation |
| Hepatobiliary Cancer | TCGA-CC-A9FS-01 | R440I | Missense_Mutation |
| Hepatobiliary Cancer | TCGA-2Y-A9H3-01 | T375S | Missense_Mutation |
| Hepatobiliary Cancer | TCGA-KR-A7K8-01 | R605P | Missense_Mutation |
| Prostate Cancer | TCGA-KK-A59X-01 | N541S | Missense_Mutation |
| Thymic Epithelial Tumor | TCGA-3S-AAYX-01 | P622Q | Missense_Mutation |
| Endometrial Cancer | TCGA-AX-A2IN-01 | K121Sfs*4 | Frame_Shift_Del |
| Endometrial Cancer | TCGA-D1-A1NZ-01 | K121Sfs*4 | Frame_Shift_Del |
| Endometrial Cancer | TCGA-EY-A549-01 | K121Sfs*4 | Frame_Shift_Del |
| Endometrial Cancer | TCGA-PG-A917-01 | K121Sfs*4 | Frame_Shift_Del |
| Endometrial Cancer | TCGA-A5-A2K7-01 | G163Efs*20 | Frame_Shift_Del |
| Endometrial Cancer | TCGA-AX-A2HG-01 | G163Efs*20 | Frame_Shift_Del |
| Endometrial Cancer | TCGA-D1-A17D-01 | G163Efs*20 | Frame_Shift_Del |
| Endometrial Cancer | TCGA-EY-A1H0-01 | G163Efs*20 | Frame_Shift_Del |
| Endometrial Cancer | TCGA-B5-A3FC-01 | P203L | Missense_Mutation |
| Endometrial Cancer | TCGA-B5-A11N-01 | K341N | Missense_Mutation |
| Endometrial Cancer | TCGA-BS-A0UJ-01 | P524S | Missense_Mutation |
| Endometrial Cancer | TCGA-D1-A103-01 | K108N | Missense_Mutation |
| Endometrial Cancer | TCGA-BK-A6W3-01 | R589* | Nonsense_Mutation |
| Endometrial Cancer | TCGA-B5-A1MX-01 | R439C | Missense_Mutation |
| Endometrial Cancer | TCGA-B5-A0JZ-01 | R684Q | Missense_Mutation |
| Endometrial Cancer | TCGA-A5-A0G2-01 | T659I | Missense_Mutation |
| Endometrial Cancer | TCGA-AP-A059-01 | P723H | Missense_Mutation |
| Endometrial Cancer | TCGA-AP-A1DK-01 | W459C | Missense_Mutation |
| Endometrial Cancer | TCGA-AP-A1DV-01 | L371I | Missense_Mutation |
| Endometrial Cancer | TCGA-AX-A06F-01 | P283H | Missense_Mutation |
| Endometrial Cancer | TCGA-AX-A1CE-01 | R116C | Missense_Mutation |
| Endometrial Cancer | TCGA-AX-A2HC-01 | P59Q | Missense_Mutation |
| Endometrial Cancer | TCGA-B5-A1MW-01 | N133Kfs*16 | Frame_Shift_Ins |
| Endometrial Cancer | TCGA-B5-A3FC-01 | D266E | Missense_Mutation |
| Endometrial Cancer | TCGA-D1-A16X-01 | Y502H | Missense_Mutation |
| Endometrial Cancer | TCGA-DI-A1BU-01 | A282V | Missense_Mutation |
| Endometrial Cancer | TCGA-EO-A3AV-01 | R161G | Missense_Mutation |
| Endometrial Cancer | TCGA-EO-A3AV-01 | F134L | Missense_Mutation |
| Endometrial Cancer | TCGA-EO-A3KX-01 | I278F | Missense_Mutation |
| Endometrial Cancer | TCGA-EY-A1GI-01 | L10F | Missense_Mutation |
| Endometrial Cancer | TCGA-QF-A5YS-01 | F5C | Missense_Mutation |
| Non-Small Cell Lung Cancer | TCGA-44-6777-01 | S385C | Missense_Mutation |
| Non-Small Cell Lung Cancer | TCGA-67-3771-01 | D266Y | Missense_Mutation |
| Non-Small Cell Lung Cancer | TCGA-62-8394-01 | G560D | Missense_Mutation |
| Non-Small Cell Lung Cancer | TCGA-05-4410-01 | S685Lfs*8 | Frame_Shift_Del |
| Non-Small Cell Lung Cancer | TCGA-17-Z060-01 | Q365H | Missense_Mutation |
| Esophagogastric Cancer | TCGA-L5-A4OJ-01 | S219C | Missense_Mutation |
| Melanoma | TCGA-FW-A3R5-06 | R511C | Missense_Mutation |
| Melanoma | TCGA-DA-A1I4-06 | H504R | Missense_Mutation |
| Melanoma | TCGA-ER-A195-06 | H504R | Missense_Mutation |
| Melanoma | TCGA-ER-A19F-06 | H504R | Missense_Mutation |
| Melanoma | TCGA-WE-AAA4-06 | H504R | Missense_Mutation |
| Melanoma | TCGA-FS-A1ZA-06 | R289* | Nonsense_Mutation |
| Melanoma | TCGA-EE-A2GI-06 | G450E | Missense_Mutation |
| Melanoma | TCGA-EB-A551-01 | I288M | Missense_Mutation |
| Melanoma | TCGA-EE-A2A2-06 | P203L | Missense_Mutation |
| Melanoma | TCGA-D3-A5GR-06 | E485* | Nonsense_Mutation |
| Melanoma | TCGA-EE-A2MD-06 | P722S | Missense_Mutation |
| Melanoma | TCGA-EE-A3AB-06 | D582G | Missense_Mutation |
| Melanoma | TCGA-D3-A2JO-06 | R603L | Missense_Mutation |
| Melanoma | TCGA-EE-A17Z-06 | S624Y | Missense_Mutation |
| Melanoma | TCGA-ER-A19P-06 | V6L | Missense_Mutation |
| Melanoma | TCGA-GN-A266-06 | G114V | Missense_Mutation |
| Melanoma | TCGA-GN-A26C-01 | G303* | Nonsense_Mutation |
| Esophagogastric Cancer | TCGA-BR-8372-01 | K121Sfs*4 | Frame_Shift_Del |
| Esophagogastric Cancer | TCGA-CD-A4MG-01 | K121Sfs*4 | Frame_Shift_Del |
| Esophagogastric Cancer | TCGA-CG-5726-01 | K121Sfs*4 | Frame_Shift_Del |
| Esophagogastric Cancer | TCGA-HF-A5NB-01 | K121Sfs*4 | Frame_Shift_Del |
| Esophagogastric Cancer | TCGA-VQ-A924-01 | K121Sfs*4 | Frame_Shift_Del |
| Esophagogastric Cancer | TCGA-BR-7851-01 | G163Efs*20 | Frame_Shift_Del |
| Esophagogastric Cancer | TCGA-CG-4465-01 | G163Efs*20 | Frame_Shift_Del |
| Esophagogastric Cancer | TCGA-VQ-A8PT-01 | T704A | Missense_Mutation |
| Esophagogastric Cancer | TCGA-CD-8536-01 | V170M | Missense_Mutation |
| Esophagogastric Cancer | TCGA-CD-8529-01 | R457T | Missense_Mutation |
| Esophagogastric Cancer | TCGA-BR-8361-01 | R372H | Missense_Mutation |
| Esophagogastric Cancer | TCGA-BR-8680-01 | M706I | Missense_Mutation |
| Esophagogastric Cancer | TCGA-F1-6874-01 | R564H | Missense_Mutation |
| Esophagogastric Cancer | TCGA-VQ-A8E3-01 | A264V | Missense_Mutation |
| Esophagogastric Cancer | TCGA-VQ-A8PT-01 | R566H | Missense_Mutation |
| Breast Cancer | TCGA-BH-A1FC-01 | R272T | Missense_Mutation |
| Breast Cancer | TCGA-BH-A0H3-01 | T421K | Missense_Mutation |
| Breast Cancer | TCGA-A2-A0ER-01 | D265Y | Missense_Mutation |
| Breast Cancer | TCGA-AQ-A7U7-01 | D476G | Missense_Mutation |
| Breast Cancer | TCGA-D8-A27V-01 | F489Lfs*2 | Frame_Shift_Del |
| Breast Cancer | TCGA-EW-A1J5-01 | Q653E | Missense_Mutation |
| Colorectal Cancer | TCGA-AD-6889-01 | K121Sfs*4 | Frame_Shift_Del |
| Colorectal Cancer | TCGA-AA-3949-01 | G616D | Missense_Mutation |
| Colorectal Cancer | TCGA-AA-3715-01 | D582V | Missense_Mutation |
| Colorectal Cancer | TCGA-AA-3852-01 | Q180* | Nonsense_Mutation |
| Colorectal Cancer | TCGA-AA-3510-01 | X246_splice | Splice_Region |
| Colorectal Cancer | TCGA-AD-5900-01 | D432G | Missense_Mutation |
| Colorectal Cancer | TCGA-AM-5821-01 | A518T | Missense_Mutation |
| Colorectal Cancer | TCGA-CA-6717-01 | E654D | Missense_Mutation |
| Colorectal Cancer | TCGA-CK-4951-01 | E368K | Missense_Mutation |
| Colorectal Cancer | TCGA-CM-6674-01 | L434P | Missense_Mutation |
| Colorectal Cancer | TCGA-WS-AB45-01 | P139L | Missense_Mutation |
| Renal Non-Clear Cell Carcinoma | TCGA-2Z-A9JK-01 | G104C | Missense_Mutation |
| Renal Non-Clear Cell Carcinoma | TCGA-B9-5155-01 | S727P | Missense_Mutation |
| Renal Non-Clear Cell Carcinoma | TCGA-WN-A9G9-01 | P700T | Missense_Mutation |
| Head and Neck Cancer | TCGA-KU-A66S-01 | G280R | Missense_Mutation |
| Head and Neck Cancer | TCGA-F7-A50J-01 | R728C | Missense_Mutation |
| Cervical Cancer | TCGA-2W-A8YY-01 | R439H | Missense_Mutation |
| Cervical Cancer | TCGA-Q1-A73O-01 | G620E | Missense_Mutation |
| Cervical Cancer | TCGA-Q1-A73O-01 | E607K | Missense_Mutation |
| Cervical Cancer | TCGA-VS-A950-01 | R372C | Missense_Mutation |
| Cervical Cancer | TCGA-2W-A8YY-01 | R684Q | Missense_Mutation |
| Pleural Mesothelioma | TCGA-TS-A7OZ-01 | G590R | Missense_Mutation |
| Seminoma | TCGA-ZM-AA0B-01 | E148D | Missense_Mutation |
